# The impact of high BMI on the global burden of osteoarthritis from 1990 to 2021 and future projections

**DOI:** 10.3389/fmed.2025.1561750

**Published:** 2025-06-18

**Authors:** Yonghui Zhao, Jiqing Wang, Boya Zhao, Yingang Zhang

**Affiliations:** ^1^Department of Orthopedics, The First Affiliated Hospital of Xi’an Jiaotong University, Xi’an, China; ^2^Center for Reproductive Medicine, The First Affiliated Hospital of Zhengzhou University, Zhengzhou, China

**Keywords:** osteoarthritis, high body mass index, knee and hip osteoarthritis, global burden of disease, health inequalities

## Abstract

**Introduction:**

Osteoarthritis (OA) is a major global cause of disability, predominantly affecting weight-bearing joints such as the knee and hip. High body mass index (BMI) is a well-established modifiable risk factor for OA. This study aimed to estimate the global burden of OA attributable to high BMI from 1990 to 2021 and to project its trends through 2036 using the Global Burden of Disease (GBD) 2021 database.

**Methods:**

We analyzed OA-related years lived with disability (YLDs) attributable to high BMI, focusing on knee and hip OA. Regional and sociodemographic disparities were evaluated using the Socio-demographic Index (SDI). Future burden trends were projected using a Bayesian Age–Period–Cohort (BAPC) model. Frontier analysis was employed to assess potential gains in health system performance.

**Results:**

From 1990 to 2021, global OA YLDs attributable to high BMI increased substantially, driven by rising obesity rates, population aging, and demographic changes. Knee OA showed the greatest increase, especially in low- and middle-SDI regions, whereas hip OA burdens were more concentrated in high-SDI regions. The BAPC model projected continued increases in YLDs through 2036. Frontier analysis revealed substantial gaps between actual and optimal OA burdens, particularly in developing regions.

**Discussion:**

Our findings highlight the growing impact of high BMI on OA burden, with pronounced regional inequalities. There is an urgent need for targeted prevention strategies, improved OA management, and optimized resource allocation, especially in low-SDI and rapidly developing areas. These results support the development of precision public health strategies to mitigate the rising burden of BMI-related OA worldwide.

## 1 Introduction

Osteoarthritis (OA) is one of the most prevalent degenerative joint diseases worldwide, closely linked to various factors such as obesity, aging, and mechanical stress, and imposes a substantial burden on both public health and socioeconomic systems (1). Knee osteoarthritis (Knee-OA) and hip osteoarthritis (Hip-OA), owing to their significant weight-bearing and mechanical loads, show a particularly pronounced association with elevated body mass index (BMI) (2). Existing evidence indicates that high BMI can markedly increase the risk of Knee-OA and Hip-OA by exacerbating joint loading and inducing low-grade systemic inflammation; furthermore, it accelerates disease progression and elevates the demand for surgical interventions and related complications (3).

Regrettably, a systematic understanding of the global and regional burden attributable to high BMI in both Knee-OA and Hip-OA remains lacking, especially with respect to the long-term trends across countries of varying Socio-Demographic Index (SDI) levels. Previous studies have largely relied on earlier releases of Global Burden of Disease (GBD) data and did not specifically focus on a single OA subtype (4, 5). To bridge these gaps, we conducted, for the first time, a simultaneous evaluation of Knee-OA and Hip-OA within a unified analytical framework using the latest GBD 2021 data. Specifically, we quantified the high BMI–related attributable risks and years lived with disability (YLDs) for knee, hip, and overall OA from 1990 to 2021. In addition, to assess the current burden, we employed Frontier Analysis to measure the discrepancy between each country’s or region’s burden and the “ideal” burden, while integrating a Bayesian Age–Period–Cohort (BAPC) model to project trends up to 2036.

Overall, this study provides a comprehensive characterization of the spatiotemporal patterns of high BMI in Knee-OA and Hip-OA, offering robust evidence for developing targeted intervention strategies and allocating resources to mitigate the future OA burden associated with high BMI. Our findings are anticipated to underscore the importance of obesity prevention and integrated OA management, while furnishing empirical insights for precision public health decision-making across countries with diverse SDI levels.

Therefore, this study aimed to examine the global, regional, and national burden of osteoarthritis (OA) attributable to high body mass index (BMI) from 1990 to 2021, and to project trends through 2036 using data from the Global Burden of Disease (GBD) 2021 study. By focusing on knee and hip OA as primary weight-bearing joints, we aimed to provide policy-relevant evidence to inform obesity prevention, OA management, and health system planning across different sociodemographic settings.

## 2 Materials and methods

### 2.1 Study data

This study was conducted using data from the GBD 2021 database, which encompasses 204 countries and regions worldwide, systematically analyzing epidemiological indicators—including incidence, prevalence, and YLDs—for various diseases and associated risk factors from 1990 to 2021 (6, 7). By employing a unified research framework and modeling approach, the GBD 2021 database provides multidimensional stratification by country/region, age, sex, and SDI, thereby offering highly comparable long-term data to facilitate cross-regional comparisons of disease burden and health metrics (8). All original data are publicly available at https://ghdx.healthdata.org/gbd-2021.

In this study, our focus was on overall OA and its two principal weight-bearing subtypes: Knee-OA and Hip-OA. The core data included the following: (1) YLDs due to OA (encompassing Knee-OA and Hip-OA) from 1990 to 2021, stratified by country/region, age, and sex; (2) attributable indicators for OA due to high BMI, including the age-standardized YLD rate (per 100,000) and its temporal trends; (3) SDI measures for individual countries and regions from 1990 to 2021; and (4) global and regional population projections for 2022–2036.

The GBD project ensures consistent definitions and estimation methods for diseases and risk factors, supported by stringent quality control and uncertainty assessments, thereby guaranteeing data comparability and reliability (9). Leveraging these high-quality, multidimensional data, we conducted a systematic evaluation of the burden of overall OA, Knee-OA, and Hip-OA attributable to high BMI at both the global and regional levels. These data also lay the groundwork for subsequent Frontier Analysis and BAPC model–based forecasts.

### 2.2 Definitions

We obtained the case definitions and estimation methods for OA from the GBD 2021 database (7). In GBD 2021, OA is defined as the most common form of arthritis, characterized by chronic inflammation, joint degeneration, and structural changes. The reference case definitions specify symptomatic knee or hip osteoarthritis radiologically confirmed as Kellgren–Lawrence grade 2–4, in individuals aged ≥ 30 years (10). Incidence, prevalence, and YLDs are reported separately for overall OA, knee OA, and hip OA. In this study, we included overall OA as well as knee OA and hip OA, given their clinical relevance as major weight-bearing joints.

High body-mass index (BMI) was defined according to the GBD 2021 risk-factor framework^1^ : for adults (≥ 20 years), BMI above the theoretical minimum-risk exposure level of 20–23 kg/m^2^, and for children and adolescents (2–19 years), overweight or obesity as classified by the International Obesity Task Force (IOTF) growth reference (11). The GBD 2021 framework quantifies the burden of OA attributable to high BMI using the Population Attributable Fraction (PAF) method, from which we extracted the attributable YLDs to evaluate the contribution of high BMI to OA overall and its subtypes. According to the GBD methodology, YLDs are calculated by multiplying disease prevalence by the appropriate disability weight and subsequently adjusting for comorbidities to derive the final estimates. As OA is primarily a disabling rather than fatal disease, our study focuses solely on the non-fatal burden of OA. All reported results are accompanied by 95% uncertainty intervals (UIs) to reflect potential variability in the data and uncertainties in the modeling process.

Additionally, we obtained SDI data for stratified analyses to explore the heterogeneous impact of high BMI on OA burden under varying levels of socioeconomic development. GBD 2021 classifies 204 countries and regions into five groups—high, high-middle, middle, low-middle, and low—based on per capita income, average years of education, and total fertility rate among women under 25 years of age. This classification supports comparability across countries for subsequent trend comparisons and resource allocation analyses.

### 2.3 Statistical analysis

We first summarized the YLDs of OA from 1990 through 2021 to estimate the Annual Percentage Change (APC) and the Estimated Annual Percentage Change (EAPC), enabling us to capture temporal trends in OA burden over this period. To elucidate the driving factors behind these trends, we performed a decomposition analysis to quantify the independent contributions of demographic shifts such as population aging, calendar-year variation, population growth, and changes in epidemiological patterns to the overall OA YLDs attributable to high BMI. By comparing a hypothetical “ideal” burden (e.g., holding one factor constant while varying others) with the observed burden, we determined the relative impact of each factor—for instance, isolating the effect of population aging to understand how age structure alters OA burden.

To examine the relationship between SDI and age-standardized YLD rates (ASYR) of OA attributable to high BMI, we calculated Pearson correlation coefficients. Moreover, we incorporated both the Slope Index of Inequality (SII) and the Concentration Index (CI) to quantify health inequalities (12). Specifically, SII captures the gradient between SDI groups, whereas CI gauges the extent to which OA burden is concentrated within a particular portion of the population. Following the rationale of Frontier Analysis, we identified the lowest level of high BMI–related OA YLDs among the highest-SDI countries as the “ideal burden.” Subsequently, we compared the disparity between that ideal burden and the actual burden observed in each of the 204 countries and regions. We highlight two sets of findings: (1) the 15 countries exhibiting the largest gap relative to the ideal OA YLDs, and (2) within the SDI < 0.5 and SDI > 0.85 strata, the five countries with the most pronounced discrepancies in each category.

We applied a Bayesian age–period–cohort (BAPC) model, implemented with the Integrated Nested Laplace Approximation (INLA) framework, to project age-standardized YLDs attributable to high BMI from 2022 to 2036. Briefly, counts of YLDs *Y*_*a,p*_ in age group *a* and calendar year *p* were assumed to follow a Poisson distribution with expected value*E_a,p_* exp(η_*a*,*p*_), where*E_a,p_* is the corresponding population exposure and


$$\eta_{a,p}=\alpha_{\alpha }+\beta_{p}+\gamma_{c},c=p-a$$

represents additive random effects for age (α_α_), period (β_*p*_), and birth cohort (γ_*c*_). Each effect was modeled as a second-order random walk with precision parameters assigned minimally informative gamma priors (shape = 0.001, rate = 0.001). Model training used Global Burden of Disease (GBD 2021) data for 1990–2021 and UN World Population Prospects 2022 population estimates; projections to 2036 incorporated the same population forecasts. Posterior means and 95 % credible intervals of age-specific rates were obtained, then age-standardized with the GBD world standard population and expressed per 100 000. All analyses were conducted in R 4.3.1 using the “BAPC” (v0.0.39) and “INLA” (v23.07.10) packages. A two-sided *P* < 0.05 defined statistical significance (13).

## 3 Results

### 3.1 Descriptive analysis of the global and regional OA YLD burden

Globally, the YLDs due to OA showed a steep increase over time, whereas the ASYR exhibited a slight upward trend from 1990 to 2021, with consistently higher values in females than males. Tables S1, S2, and S3 present the numbers, ASYR, and estimated annual percentage changes (EAPCs) for OA-related YLDs in 1990 and 2021. Regionally, East Asia had the highest OA YLDs, while North America (high-income countries) showed relatively lower ASYR (Supplementary Tables S1–S3).

### 3.2 Spatiotemporal distribution of high BMI–Related OA YLDs and decomposition of trends

**FIGURE 1:**
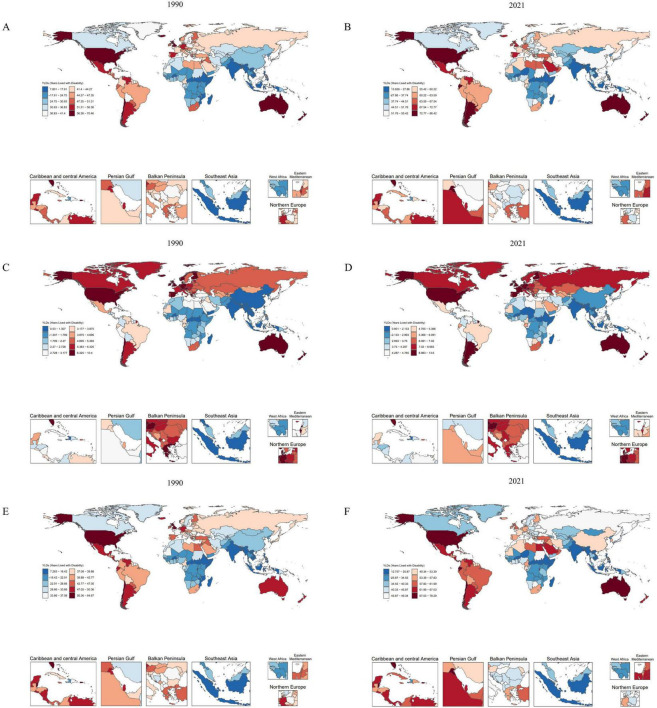
Comparison of age-standardized YLD rates (per 100 000) in 204 countries and regions in 1990 and 2021. **(A,B)** Age-standardized YLD rates (per 100,000) attributable to high BMI in OA for 1990 and 2021. **(C,D)** Age-standardized YLD rates (per 100,000) attributable to high BMI in Hip-OA for 1990 and 2021. **(E,F)** Age-standardized YLD rates (per 100,000) attributable to high BMI in Knee-OA for 1990 and 2021. BMI, body mass index; OA, osteoarthritis; Hip-OA, hip osteoarthritis; Knee-OA, knee osteoarthritis.

As illustrated in Figure 1, from 1990 to 2021, the global burden of OA—including Hip-OA and Knee-OA—attributable to elevated BMI increased substantially, with Knee-OA experiencing the most prominent surge. This upward trend was predominantly driven by rising obesity prevalence, population growth, and population aging, particularly in regions with low and middle SDI. High-SDI areas also faced a notable disease burden, largely due to higher obesity rates and an aging population.

**FIGURE 2:**
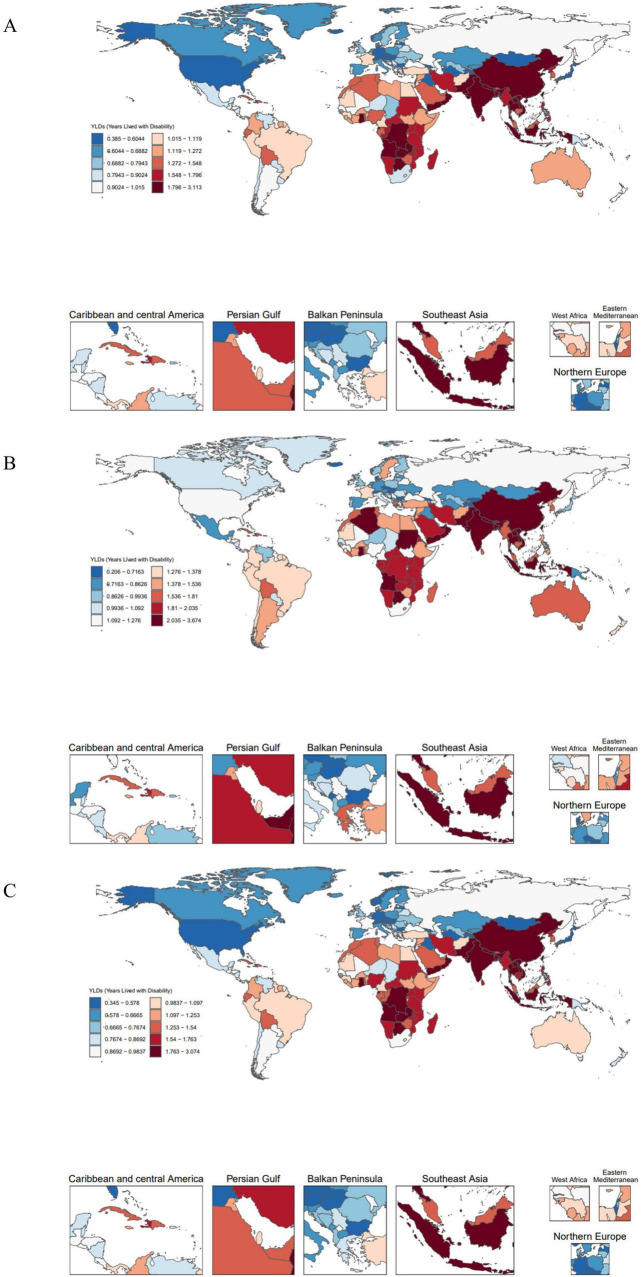
Global distribution of EAPC in 204 countries. **(A)** EAPC of OA age-standardized YLD rates (per 100,000) attributable to high BMI. **(B)** EAPC of Hip-OA age-standardized YLD rates (per 100,000) attributable to high BMI. **(C)** EAPC of Knee-OA age-standardized YLD rates (per 100,000) attributable to high BMI. EAPC, estimated annual percentage change; BMI, body mass index; OA, osteoarthritis; Hip-OA, hip osteoarthritis; Knee-OA, knee osteoarthritis.

**FIGURE 3:**
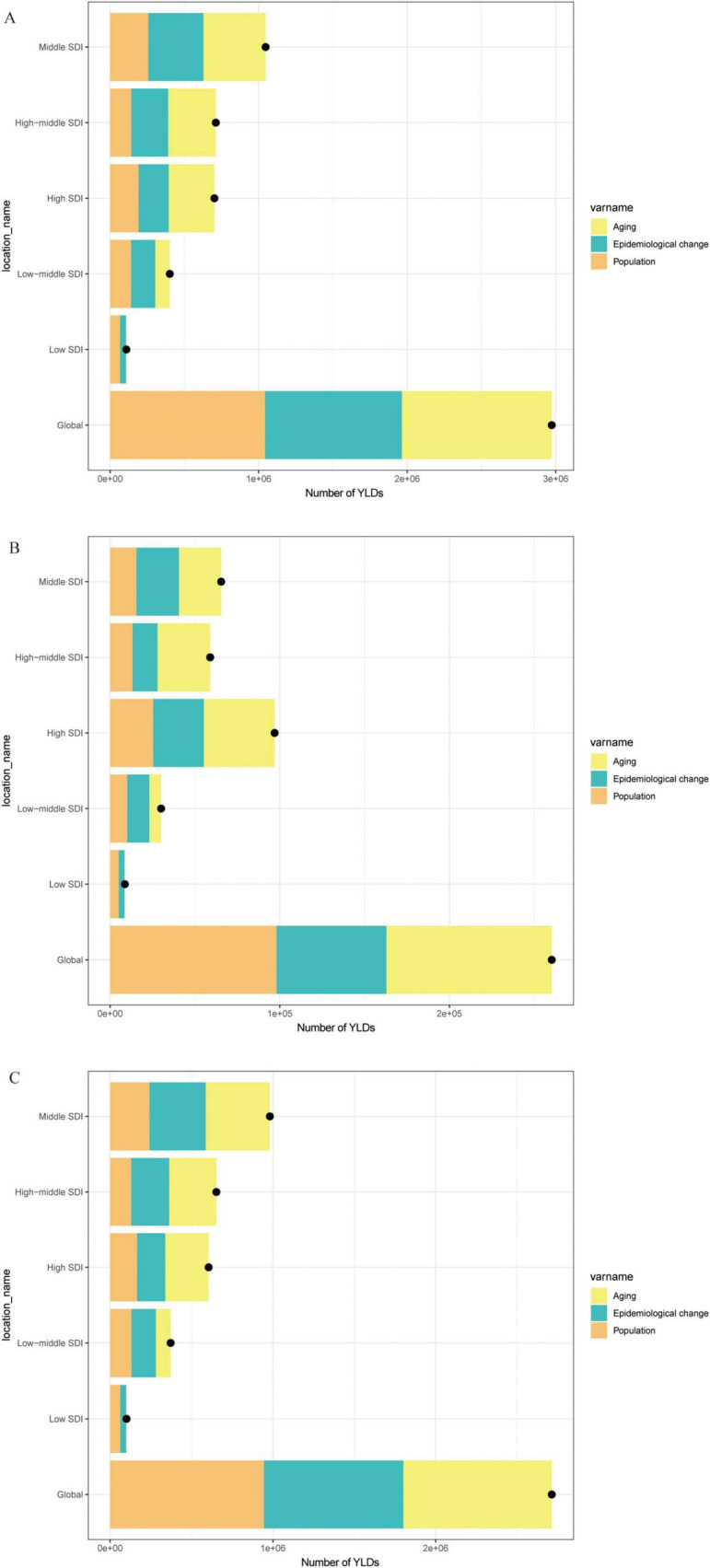
Decomposition analysis of trends in the number of YLDs, 1990–2021. **(A)** Number of YLDs attributable to high BMI for OA. **(B)** Number of YLDs attributable to high BMI for Hip-OA. **(C)** Number of YLDs attributable to high BMI for Knee-OA. BMI, body mass index; OA, osteoarthritis; Hip-OA, hip osteoarthritis; Knee-OA, knee osteoarthritis.

The global map of EAPCs (Figure 2) indicates that South Asia, Southeast Asia, and parts of Africa experienced the fastest growth over the past three decades, with Knee-OA showing the largest increase and Hip-OA displaying a comparatively smaller rise. Our decomposition analysis (Figure 3) further reveals that from 1990 to 2021, the overall increase in OA burden due to high BMI was chiefly propelled by population growth and aging, with Knee-OA growing most strikingly in low- and middle-SDI regions. In high-SDI areas, aging represented the principal driver, whereas in lower-SDI settings, population expansion was the main factor. The impact of epidemiological shifts varied across regions: in some regions, the escalating prevalence of high BMI exacerbated the disease burden, whereas in certain high-SDI countries, effective health management and medical interventions partially mitigated these adverse effects.

### 3.3 Relationship with SDI, sex, and age

**FIGURE 4:**
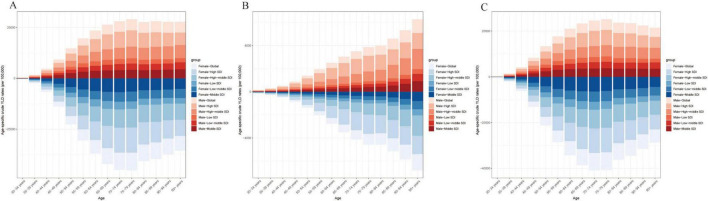
Age-specific crude YLD rates attributable to high BMI by SDI region, 1990–2021. **(A)** Trends in age-specific crude OA YLD rates (per 100 000) across SDI regions. **(B)** Trends in age-specific crude Hip-OA YLD rates (per 100 000) across SDI regions. **(C)** Trends in age-specific crude Knee-OA YLD rates (per 100,000) across SDI regions. BMI, body mass index; OA, osteoarthritis; Hip-OA, hip osteoarthritis; Knee-OA, knee osteoarthritis.

**FIGURE 5:**
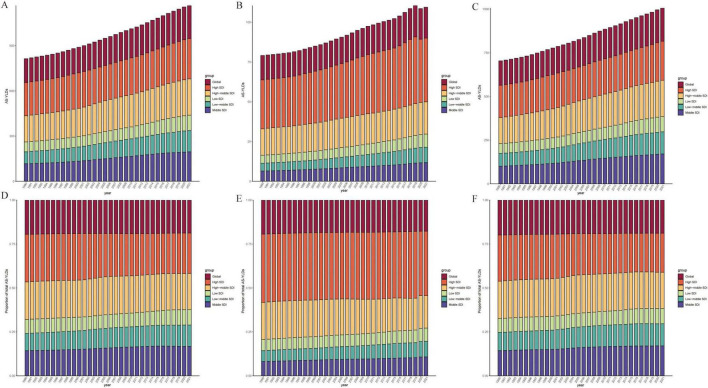
Time-Trend Analysis of High BMI–Related age-standardized YLD rates (per 100,000). **(A–C)** Age-standardized YLD rates (per 100,000) attributable to high BMI from 1990 to 2021 for OA **(A)**, Hip-OA (B), and Knee-OA (C) across six SDI regions (Global, High, High-middle, Middle, Low-middle, and Low SDI). **(D–F)** Proportion of the total age-standardized YLD rates (per 100,000) contributed by each SDI region over time for OA **(D)**, hip OA **(E)**, and knee OA **(F)**. The x-axis represents calendar year (1990–2021), and the y-axis shows either the age-standardized YLD rates (per 100,000) or the percentage contribution to the total. BMI, body mass index; AS-YLDs, age-standardized YLD rates (per 100,000); OA, osteoarthritis; Hip OA, hip osteoarthritis; Knee OA, knee osteoarthritis.

As shown in Figure 4, an age-specific trend analysis indicates that the crude YLDs of OA and its subtypes increase markedly with advancing age, particularly in individuals aged 60 years or older. In high-SDI areas, the burden is primarily concentrated among older adults, and females consistently experience higher crude YLDs than males, especially for Hip-OA. By contrast, Knee-OA poses a more pronounced burden in low- to middle-SDI settings, affecting both middle-aged and older populations (≥ 40 years), with higher YLD rates. Figure 5 illustrates the temporal trends in OA, Hip-OA, and Knee-OA age-standardized YLDs attributable to high BMI from 1990 to 2021, stratified by SDI regions. The results demonstrate a persistent upward trend across all SDI levels. Notably, OA and Hip-OA have increased most sharply in high- and high-middle-SDI areas, whereas Knee-OA has surged most rapidly in low- and middle-SDI areas.

**FIGURE 6:**
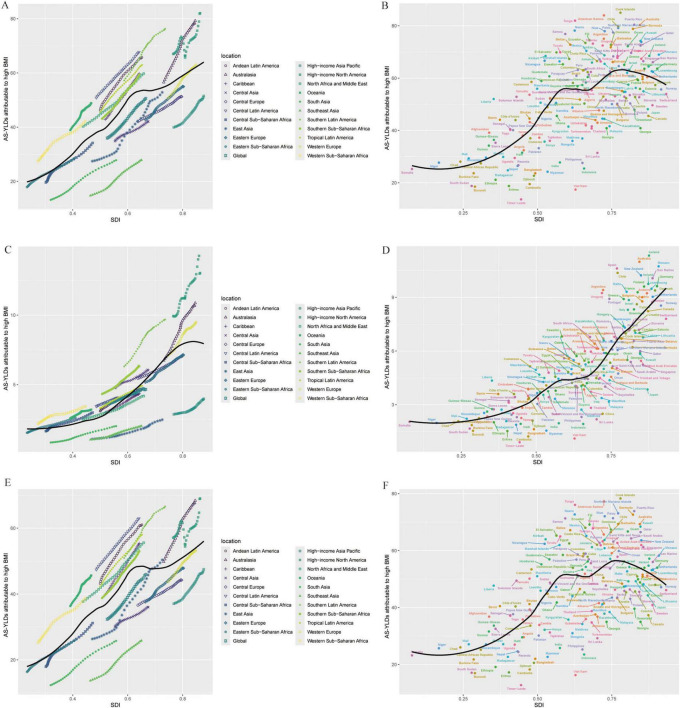
Relationship between OA age-standardized YLD rates (per 100,000) and SDI. **(A,B)** Age-standardized YLD rates (per 100,000) for OA: across SDI levels in different global regions **(A)**; across 204 countries **(B)**. **(C,D)** Age-standardized YLD rates (per 100,000) for Hip-OA: across SDI levels in different global regions **(C)**; across countries **(D)**. **(E,F)** Age-standardized YLD rates (per 100,000) for Knee-OA: across SDI levels in different global regions **(E)**; across countries **(F)**. The x-axis represents the SDI, and the y-axis represents the age-standardized YLD rates (per 100,000). Different colors indicate SDI regions: Global, High SDI, High-middle SDI, Middle SDI, Low-middle SDI, and Low SDI. BMI, body mass index; AS-YLDs, age-standardized YLD rates (per 100,000); OA, osteoarthritis; Hip-OA, hip osteoarthritis; Knee-OA, knee osteoarthritis.

**FIGURE 7:**
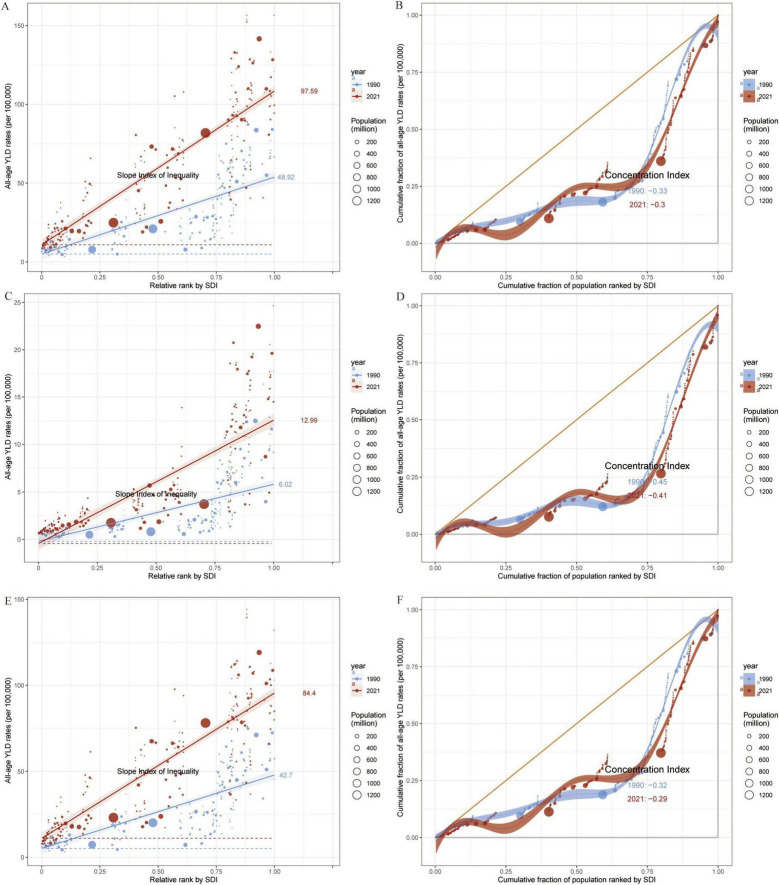
Slope Index and Concentration Index for Global all-age YLD rates (per 100,000), 1990–2021. **(A,B)** All-age YLD rates (per 100,000) for OA attributable to high BMI: Slope Index of Inequality **(A)** and Concentration Index **(B)** across 204 countries. **(C,D)** All-age YLD rates (per 100,000) for Hip-OA attributable to high BMI: Slope Index of Inequality **(C)** and Concentration Index **(D)** across 204 countries. **(E,F)** All-age YLD rates (per 100,000) for Knee-OA attributable to high BMI: Slope Index of Inequality **(E)** and Concentration Index **(F)** across 204 countries. Different colors indicate SDI regions: Global, High SDI, High-middle SDI, Middle SDI, Low-middle SDI, and Low SDI. BMI, body mass index; OA, osteoarthritis; Hip-OA, hip osteoarthritis; Knee-OA, knee osteoarthritis.

Figure 6 further depicts the association between age-standardized YLDs and SDI, revealing a significant rise in all OA types with increasing SDI. High-SDI countries (e.g., in North America, Western Europe, and Oceania) bear the highest burden, although regions with low SDI show lower absolute values that are nevertheless increasing over time. The strongest positive correlation with SDI emerges for Knee-OA, particularly in high-SDI countries, where growth is especially notable. Hip-OA remains more concentrated in economically developed countries, and its burden in low-SDI regions is comparatively limited. Similarly, Figure 7 demonstrates that the age-standardized YLDs of all OA subtypes escalate significantly with rising SDI, peaking in high-SDI regions (such as North America, Western Europe, and Oceania). Although low-SDI areas exhibit lower absolute values, they have also experienced incremental growth over time. Among these patterns, Knee-OA shows the most pronounced positive correlation with SDI, with high-SDI countries showing a particularly steep increase; Hip-OA, on the other hand, remains more geographically focused in developed economies, with relatively limited burdens observed in low-SDI areas.

### 3.4 Health inequality analysis

Results based on the SII and CI (Figure 7) uncover the association between all age OA YLDs attributable to high BMI and SDI from 1990 to 2021. The findings indicate that the overall all age YLDs of OA and its subtypes increase with SDI, with the highest burdens observed in high-SDI regions, especially in high-income areas with pronounced aging. By contrast, low- to middle-SDI regions, despite having a lower current burden, are experiencing a faster rate of increase, particularly for Knee-OA, which exceeds Hip-OA burden in these populations.

### 3.5 Frontier analysis of ideal burden and projections to 2050

**FIGURE 8:**
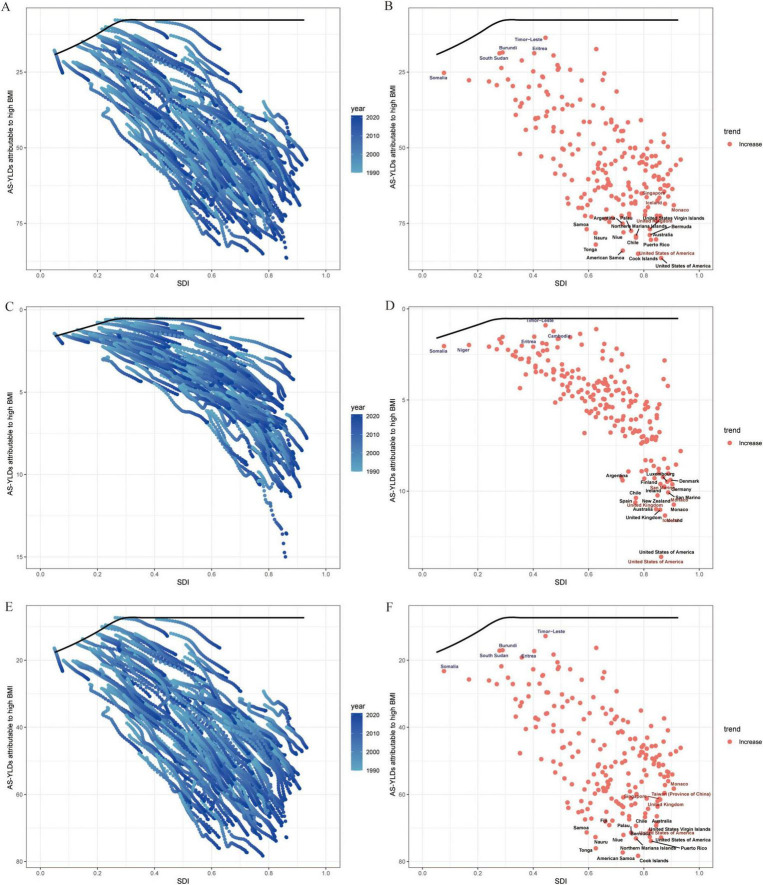
Frontier analysis of SDI and OA age-standardized YLD rates (per 100,000) in 204 countries and regions. **(A)** Trajectories of OA age-standardized YLD rates (per 100,000) in 204 countries from 1990 to 2021; line colors fade from light to dark blue with advancing calendar year. The black curve marks the frontier—the lowest burden observed at each level of the Sociodemographic Index (SDI). **(B)** Country-level OA age-standardized YLD rates (per 100,000) in 2021 plotted against the same frontier. **(C,D)** Corresponding plots for Hip-OA. **(E,F)** Corresponding plots for Knee-OA. The x-axis represents the Sociodemographic Index (SDI, 0–1), and the y-axis represents age-standardized YLD rates (per 100,000). BMI, body mass index; AS-YLDs, age-standardized YLD rates (per 100,000); OA, osteoarthritis; Hip-OA, hip osteoarthritis; Knee-OA, knee osteoarthritis.

In the frontier analysis, we used the lowest age standardized YLDs among the highest-SDI countries as the “ideal burden” and compared the gaps between each country’s burden and this ideal value. The results suggest that high-SDI countries are generally closer to the efficiency frontier, whereas low- to middle-SDI countries deviate substantially, indicating ample room for improvement in disease intervention and management. Even among high-SDI countries, Hip-OA burdens vary significantly; however, Knee-OA divergence is more striking in low- to middle-SDI areas (Figure 8).

**FIGURE 9:**
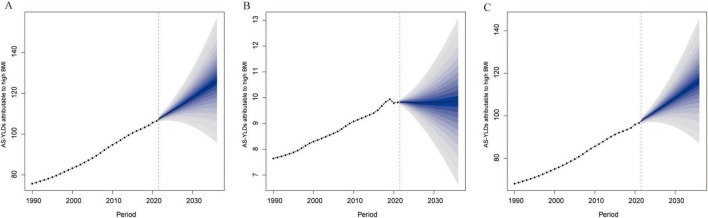
BAPC projections of High BMI–related age-standardized YLD rates (per 100,000) to 2036. **(A)** Projected OA age-standardized YLD rates (per 100,000) due to high BMI based on the Bayesian Age–Period–Cohort (BAPC) model. **(B)** Projected Hip-OA age-standardized YLD rates (per 100,000) due to high BMI based on the BAPC model. **(C)** Projected knee-OA age-standardized YLD rates (per 100,000) due to high BMI based on the BAPC model. The x-axis represents the year, and the y-axis represents age-standardized YLD rates (per 100,000). BAPC, Bayesian age–period–cohort; BMI, body mass index; AS-YLDs, age-standardized YLD rates (per 100,000); OA, osteoarthritis; Hip-OA, hip osteoarthritis; Knee-OA, knee osteoarthritis.

Projections based on the BAPC model (Figure 9) indicate that the global burden of OA and its subtypes attributable to high BMI will continue to grow through 2036. Knee-OA is expected to register the most substantial increase, likely emerging as a predominant public health challenge, while Hip-OA, although lower in absolute terms, also exhibits a steady upward trend. Notably, the relatively narrow uncertainty intervals suggest robust reliability for these forecasts.

## 4 Discussion

Drawing on the GBD 2021 database, we conducted a comprehensive analysis of the global disease burden attributable to high BMI in OA (including hip and knee subtypes) from 1990 to 2021. Our findings show that the OA burden related to high BMI has increased significantly over the past three decades and exhibits substantial disparities across SDI regions (14). Notably, the overall burden of OA, including its hip and knee subtypes, is relatively higher in upper-middle and high SDI regions, while in low SDI regions—where population growth and aging are accelerating—its absolute growth also warrants attention. Compared with previous studies that focused on single OA subtypes or specific regions, our work integrates three types of OA (overall OA, Hip-OA, and Knee-OA) on a global scale, offering critical insights into the comprehensive impact of obesity on OA burden.

Consistent with earlier studies (15), we observed that the effects of high BMI on OA vary by socioeconomic status and population susceptibility. Specifically, medium-SDI countries contribute a substantial share of the total YLDs of OA attributable to high BMI, and although low-SDI regions currently shoulder a lower disease burden, their age-standardized burdens continue to rise due to shifting population structures. Echoing prior GBD analyses (16, 17), obesity is playing an increasingly prominent role in a range of chronic noncommunicable conditions, disproportionately affecting women and middle-aged or older adults. Moreover, similar to other relevant studies (18), our results indicate that with rapid economic development and lifestyle changes, obesity-related OA burdens continue to climb in regions such as North America, Western Europe, and Oceania. Meanwhile, in certain parts of Asia and Africa, although the overall burden remains comparatively low, it is growing at a rapid pace.

It is worth noting that our findings also differ from prior literature in some respects. Some studies suggest that in certain high-income countries, early OA progression may be slowing due to weight management, increased physical activity, and screening efforts (19, 20). However, according to our decomposition results, the EAPCs for Hip-OA and Knee-OA remain positive in many high-SDI countries, implying that epidemiological shifts (e.g., further rises in obesity prevalence and population aging) could continue to exacerbate disease burdens. Additionally, the attributable contribution of high BMI, compared with other potential risk factors (e.g., joint injury, inflammatory pathways, genetic predisposition), varies across regions and population subgroups and demands further investigation. In low-SDI areas, the combination of surging BMI and population expansion indicates that the disease burden may escalate considerably in the future.

Overall, our study demonstrates a marked global rise in OA attributable to high BMI over the past 30 years, along with pronounced heterogeneity across SDI regions. Several factors could account for this trend. First, obesity not only increases mechanical stress on joints, hastening cartilage wear, but also triggers low-grade systemic inflammation that elevates OA onset and progression risk (21, 22). Second, with accelerating socioeconomic development and urbanization, reductions in physical activity, along with unhealthy dietary patterns, further contribute to increasing obesity prevalence (23). In many low-SDI countries with large and rapidly growing populations, the future OA burden may expand more drastically (24).

In comparing different SDI regions, we found that Knee-OA is increasing more prominently in high-SDI countries, while Hip-OA remains substantial among high-income populations. Such variations might reflect differential access to healthcare resources, early diagnosis and treatment, the demand for joint replacement surgeries, as well as an accumulated older adult population due to extended life expectancy (25). In low-SDI settings, population expansion is the key driver of future risk, emphasizing the need for early intervention in obesity prevention to mitigate the looming disease burden. Moreover, as highlighted by our health inequality analysis, Knee-OA YLDs in low- and middle-SDI regions are growing more rapidly and exceed those for Hip-OA, underscoring inadequacies in lifestyle interventions, healthcare coverage, and obesity prevention strategies.

Regarding sex differences, female patients consistently show higher OA YLDs than males, aligning with previous reports (26, 27). Biologically, this may stem from differences in body fat distribution and hormonal profiles (28), while Racial Factors could also play a role (29). Age-stratified analyses further indicate that disease burden escalates most steeply in individuals aged ≥ 50 years, suggesting that targeted obesity management and joint care initiatives should focus on older adults.

Despite the robustness of our analysis, several limitations merit discussion. First, due to limited data coverage in some regions, especially in low-SDI countries, GBD estimates may carry some degree of model-based uncertainty (30). Second, although we focused on high BMI—a key modifiable risk factor for OA—other etiological contributors such as genetic susceptibility, occupational exposures, and osteoporosis could interact in ways that are not fully captured here (31–33). Third, while our application of the BAPC model and frontier analysis is standard for disease forecasting and efficiency assessments, their accuracy and generalizability remain subject to the quality of historical data and uncertainties about future policy interventions (34).

This study has several strengths. First, it provides the most recent and comprehensive assessment of global OA burden attributable to high BMI using GBD 2021 data, covering both historical trends and future projections through 2036. Second, the analysis differentiates between knee and hip OA, which are clinically and epidemiologically distinct yet highly impactful. Third, the use of Bayesian Age–Period–Cohort modeling and frontier analysis offers novel insights into disease trajectory and health system performance, identifying gaps between current and attainable burden levels.

In conclusion, the burden of OA attributable to high BMI has been rising steadily worldwide, with particularly sharp increases in high-SDI countries and certain emerging economies; at the same time, low-SDI regions also face a considerable potential for future growth. Leveraging the latest GBD data and multiple statistical models, our study underscores the pivotal role of obesity in OA onset, progression, and socioeconomic consequences, providing critical evidence for healthcare resource allocation and public health policies. Based on our trend forecasts, in the absence of effective interventions, the OA burden associated with high BMI is likely to continue climbing in the coming decades, potentially evolving into a major public health challenge for some nations. Therefore, we recommend increased attention and investment in obesity prevention policies, joint health education, early screening, and multidisciplinary interventions—particularly for women and older adults. By enhancing international collaboration and sharing best practices, we may effectively curb the rising global trend of high BMI–related OA, thereby safeguarding population health and socioeconomic development worldwide.

## 5 Conclusion

Our study demonstrates a marked global rise in OA attributable to elevated BMI over the past three decades, with pronounced disparities across different SDI levels. This disease burden is particularly salient in individuals aged 50 years and above as well as in women, underscoring the importance of targeted prevention and management strategies. The findings highlight the urgent need to prioritize obesity control and OA prevention in public health agendas, especially in low- and middle-SDI regions. Strengthening early screening efforts, promoting healthy lifestyles, and ensuring equitable access to healthcare resources may help mitigate the continuous escalation of obesity-related OA worldwide.

## Data Availability

Publicly available datasets were analyzed in this study. This data can be found at: https://www.healthdata.org.

## Footnotes

1
https://www.healthdata.org/research-analysis/diseases-injuries-risks/factsheets/2021-high-body-mass-index-level-2-risk

## Supplementary material

Supplementary Table S1 Number, age-standardized rate (ASR), and estimated annual percentage change (EAPC) of OA YLDs in men and women in 1990 and 2021, stratified by SDI quintiles and GBD regions.

Supplementary Table S2 Number, age-standardized rate (ASR), and estimated annual percentage change (EAPC) of Hip-OA YLDs in men and women in 1990 and 2021, stratified by SDI quintiles and GBD regions.

Supplementary Table S3 Number, age-standardized rate (ASR), and estimated annual percentage change (EAPC) of Knee-OA YLDs in men and women in 1990 and 2021, stratified by SDI quintiles and GBD regions.
